# Mid-term outcomes of the sutureless marsupialization technique for acquired pulmonary vein stenosis and occlusion

**DOI:** 10.1007/s11748-025-02253-9

**Published:** 2026-01-13

**Authors:** Hironari Shibahara, Hideki Ito, Shinichi Ashida, Tomo Yoshizumi, Sachie Terazawa, Yoshiyuki Tokuda, Yuji Narita, Hajime Sakurai, Masato Mutsuga

**Affiliations:** 1 Department of Cardiovascular Surgery, Ogaki Municipal Hospital, 86-4 Minaminokawa-Cho, Ogaki City, Gifu Prefecture 503-8502 Japan; 2 Department of Cardiovascular Surgery, Tosei General Hospital, Seto, Aichi Japan; 3 Department of Cardiac Surgery, Nagoya University Graduate School of Medicine, Nagoya, Japan

**Keywords:** Catheter ablation, Pulmonary vein stenosis, Pulmonary vein occlusion, Sutureless marsupialization technique

## Abstract

**Objective:**

Pulmonary vein stenosis is a rare but serious complication following catheter ablation for atrial fibrillation. This study aimed to evaluate the mid-term outcomes of the sutureless marsupialization technique for acquired pulmonary vein stenosis or pulmonary vein occlusion.

**Methods:**

Between 2006 and 2024, six patients (mean age: 54.5 ± 9.0 years) with severe pulmonary vein stenosis or pulmonary vein occlusion after catheter ablation underwent surgical repair using the sutureless marsupialization technique. This approach avoids direct suturing to the pulmonary vein wall by covering the opened vein with autologous or xenogeneic tissue (left atrial appendage, pericardium, or atrial wall). A total of 13 pulmonary veins were reconstructed. Restenosis was evaluated using follow-up computed tomography, and 5-year patency was estimated by Kaplan–Meier analysis.

**Results:**

All patients underwent successful repair without perioperative complications. Covering materials included the left atrial appendage (n = 3), bovine pericardium (n = 2), autologous pericardium (n = 1), and atrial wall flap (n = 1). During a mean follow-up of 62.5 ± 46.5 months, restenosis occurred in 2 of 13 veins (15.4%) four months after surgery, both initially classified as stenotic lesions. All patients remained asymptomatic and required no further intervention. The 5-year patency rate was 84.6%.

**Conclusions:**

The sutureless marsupialization technique offers good mid-term outcomes for acquired pulmonary vein stenosis and pulmonary vein occlusion after catheter ablation. By avoiding direct vein wall suturing, this approach may reduce restenosis. These results support its potential as a surgical option in selected patients with this rare complication.

## Introduction

Whether or not clinical symptoms are present, a certain percentage of patients who have undergone catheter ablation (CA) experience pulmonary vein stenosis (PVS) caused by collagen replacement at the site of myocardial injury from the energy device [1, 2].

The primary symptoms include respiratory complaints such as cough and dyspnea [1, 3]. PVS typically manifests three to six months after radiofrequency catheter ablation (RFCA), and the presence and severity of symptoms depend on the number of affected vessels and the degree of stenosis. In general, treatment is indicated when the stenosis is severe and symptomatic. Early intervention is recommended for the recovery of pulmonary blood flow. However, if intervention to the PVS is delayed, there has been a report that lung resection may be considered [4].

Endovascular therapy, such as balloon angioplasty with or without stenting, is often chosen as the first-line treatment method, but its problem is a high restenosis rate. One study reported a 3-year restenosis rate after catheter treatment of 37% [3]. Surgical repair methods, such as artificial vessel replacement and patch formation, which have already been reported, also suffer from high pulmonary vein restenosis rates, reported to be 38% [5]. It has been reported that direct suture to the pulmonary vein wall can cause neo-intima formation, leading to stenosis [6]. We therefore devised a marsupialization technique in which the stenosis is covered by the left atrial appendage wall or pericardium, without directly suturing the pulmonary vein wall, and reported its good result previously [7]. We continued to treat more cases, and to date, have treated six cases similarly. This is the first report of the mid-term results of the sutureless marsupialization technique for PVS after CA for atrial fibrillation (Afib).

### Objective

In this study, the objective was to evaluate the mid-term outcomes of the sutureless marsupialization technique for acquired PVS or pulmonary vein occlusion (PVO).

## Methods

### Ethics statement

The study was approved by Ogaki Municipal Hospital (No. 20250626-26) and complies with the Helsinki Declaration. The need for patient informed consent was waived based on the retrospective nature of the study.

### Patient population

From May 2006 to July 2024, nine patients diagnosed with PVS or PVO following CA for Afib underwent surgical treatment at our institution, performed by two experienced cardiovascular surgeons. Three of these cases were excluded because they underwent surgical repairs other than the sutureless marsupialization technique, such as pulmonary vein primary anastomosis and autologous pericardial roll graft. Therefore, the remaining six cases were analyzed.

**Fig. 1:**
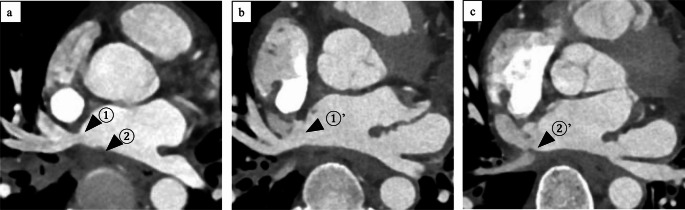
Example showing pulmonary vein stenosis before and after surgery. **a** Preoperative CT shows severe stenosis of the RMPV (black arrowhead ①) and obstruction of the RIPV (black arrowhead ②). **b**, **c** Postoperative CT shows dilation of the RMPV (black arrowhead ①’) and patency of the RIPV (black arrowhead ②’). CT: computed tomography, RIPV: right inferior pulmonary vein, RMPV: Right middle pulmonary vein

In all patients who underwent surgical treatment at our hospital, the degree of stenosis was assessed preoperatively using computed tomography (CT) (Fig. 1A). Post-ablation CT imaging was not routinely performed; in most cases, CT evaluation was conducted when symptoms such as hemoptysis or dyspnea appeared. PVS was defined as a reduction in pulmonary vein (PV) diameter on post-ablation CT compared with pre-ablation CT, and it was classified as mild (< 50%), moderate (50–70%), or severe (≥ 70%) [8]. Complete occlusion (100%) was diagnosed when no contrast enhancement was observed in the distal portion of the pulmonary veins on contrast-enhanced CT. After the surgery, CT was again performed to evaluate the patency of reconstructed veins (Fig. 1B, C) and follow-up CT examinations were routinely conducted at 1 week, 3 months, 6 months, and 12 months postoperatively. Restenosis was defined as any progression of stenosis or occlusion on immediate postoperative or follow-up CT, compared with pulmonary vein diameter on preoperative CT.

### Surgical technique

**Fig. 2:**
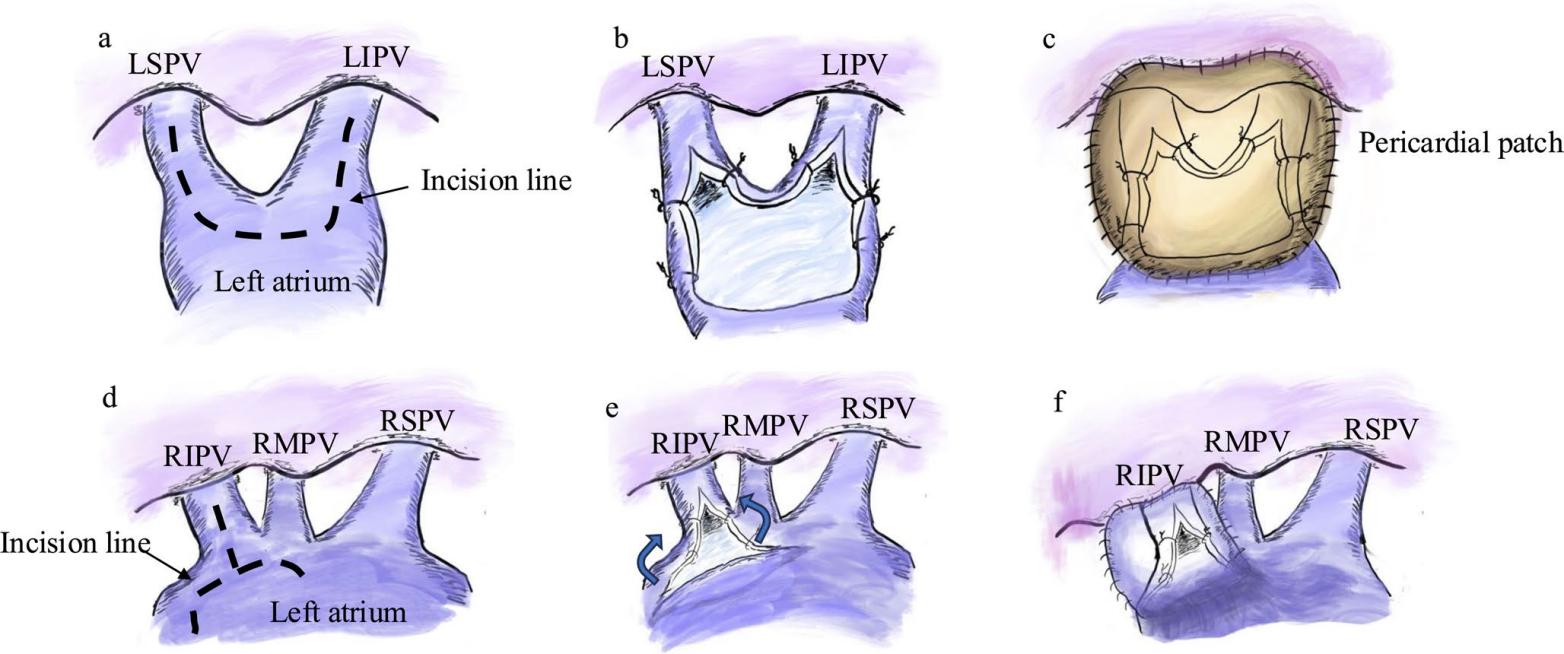
Sutureless marsupialization technique with a pericardial patch on left pulmonary vein lesions (**a**–**c**) or an atrial wall flap on right pulmonary vein lesions (**d**, **e**). The technique with the left atrial appendage has been described in a previous report [7]. **a** The pulmonary veins are incised beyond the stenosis. **b** A stay suture is applied to keep the orifice open. **c** The PV orifice is covered with the pericardial patch. **d** The pulmonary vein and left atrial wall are incised. **e**, **f** The PV orifice is covered with a flap created by the atrial wall flap. LIPV: left inferior pulmonary vein, LSPV: left superior pulmonary vein, RIPV: right inferior pulmonary vein, RMPV: right middle pulmonary vein, RSPV: right superior pulmonary vein

As described in our previous report, the surgical procedure was performed by median sternotomy, and cardiopulmonary bypass was established using bicaval cannulation and ascending aortic perfusion, followed by cardiac arrest achieved with antegrade cardioplegic perfusion [7]. When the right superior pulmonary vein (RSPV) was not involved, the left ventricular vent was inserted through the RSPV as in standard practice. However, in cases with concomitant the RSPV lesion, the vent was inserted through the anastomotic site after completing the right pulmonary vein reconstruction. Moderate hypothermia (nasopharyngeal temperature 25 °C) was applied to obtain a bloodless operative field by controlling blood flow. The venoatrial junction was exposed using a heart net, with the left side in particular being exposed by rotating the heart in a counterclockwise direction. *Backflow from the opened pulmonary veins was controlled using a cardiotomy suction cannula*. In the previously reported case, the sutureless marsupialization technique was performed using the left atrial appendage [7]; however, in the six cases in the present study, pericardial material such as autologous pericardium or bovine pericardium and an atrial wall flap were also used (Fig. 2). For only the left-sided lesions, the left atrial appendage was basically used [7]. Pericardial materials were used to cover the orifice in the cases with a small left atrial appendage. In cases of bilateral lesions, particularly on the right side where the left atrial appendage is absent, alternative materials such as pericardial material or an atrial wall flap were used to facilitate sutureless marsupialization (Fig. 2).

As we previously reported, the sutureless technique was applied without direct suturing to the pulmonary vein wall, but rather by suturing to cover the opened pulmonary vein. The suture line was anchored proximally to the left atrial wall and distally to the pericardium surrounding the pulmonary vein. When materials other than the left atrial appendage were used, the suture line was similarly extended from the left atrium–pulmonary vein junction to the pericardium to ensure coverage of the opened pulmonary vein (Fig. 2). In addition, in the present study, therapeutic intervention was performed for cases with total pulmonary vein occlusion on preoperative CT. Even if preoperative CT showed complete interruption of pulmonary venous flow, surgical repair was performed intraoperatively if the occluded lesion was incised longitudinally toward the peripheral side, and the vessel lumen was identified.

### Statistical analysis

All analyses were performed with EZR R 4.4.1. Categorical data are presented as percentages and numbers, and continuous data are presented as mean and standard deviations. The rate of 5-year freedom from PV restenosis was estimated by the Kaplan–Meier method.

## Results

The study group consisted of six male patients with a mean age of 54.5 ± 9.0 years who underwent surgical intervention for PVS after CA. Arrhythmia types were mainly AFib (n = 6), and the types of CA included radiofrequency ablation in 4 cases (66.7%), cryoablation in 1 case (16.7%), and hot balloon ablation in 1 case (16.7%). The mean time to diagnosis since the last CA was 19.7 ± 16.6 months, and the average number of ablation procedures was 2.5 ± 1.2 times (Table 1). The main symptom of the six patients was hemoptysis (N = 3) (Table 1).

**Table 1:** Patients’ preoperative characteristics

Patient	Age (y)	Sex	Symptom	Ablation type	Number of ablations	Time to diagnosis (months)
1	59	Male	No	Radiofrequency	3	26
2	63	Male	Hemoptysis	Radiofrequency	3	8
3	43	Male	No	Radiofrequency	4	50
4	65	Male	Cough	Hot balloon	1	18
5	48	Male	Hemoptysis	Radiofrequency	1	9
6	49	Male	Hemoptysis	Cryoablation	3	7
Mean ± SD	54.5 ± 9.0	–	–	–	2.5 ± 1.2	19.7 ± 16.6

The six patients had a total of 14 PVS lesions, and 13 were reconstructed surgically. The distribution of the 14 PVS lesions was as follows: RSPV in two (14.3%), right middle pulmonary vein (RMPV) in one (7.1%), right inferior pulmonary vein (RIPV) in three (21.4%), left superior pulmonary vein (LSPV) in five (35.7%), and left inferior pulmonary vein (LIPV) in three (21.4%) (Table 2). All pulmonary veins that underwent therapeutic intervention were cases of severe pulmonary vein stenosis with ≥ 70% luminal narrowing. Complete occlusion was observed in six (42.9%), with severe stenosis in eight (57.1%) (Table 2). One of 14 PVs could not be reconstructed due to an unconfirmed lumen (Table 2, Patient No. 6, RSPV). Of the six cases, five were elective surgeries, and one was emergency surgery (Patient No. 1). This case involved stent placement in the catheterization room for PVS. During the procedure, the pulmonary vein was injured, leading to hemorrhagic shock, which required emergency surgery for repair.

**Table 2:** Detailed preoperative and postoperative statuses of patients and pulmonary vein stenosis (PVS) lesions

Patient	Right side		Left side		Follow-up (months)
	Surgical material	SPV	Surgical material	SPV	
		MPV		╲	
		IPV		IPV	
1	╲		Autologous pericardium	◎ → ◯	145
			╲		
2	╲		Left appendage	● → ◯	66
			╲		
	Atrial wall	◎ → ◎	Left appendage	◎ → ◎	
3	╲		Left appendage	◎ → ◯	64
			╲		
			Left appendage	● → ◯	
4	Bovine pericardium	◎ → ◯	╲		63
	╲				
	Bovine pericardium	● → ◯			
5	╲		Left appendage	◎ → ◯	27
			╲		
6	╲		Bovine pericardium	● → ◯	10
	Bovine pericardium	◎ → ◯	╲		
	Bovine pericardium	● → ◯	Bovine pericardium	◎ → ◯	

*CT* computed tomography, *IPV* inferior pulmonary vein, *MPV* middle pulmonary vein, *SPV* superior pulmonary vein

PVS lesions on preoperative CT are described in the table as follows: patent ◯, stenosis ◎, occlusion ●

Postoperative restenosis was defined as either a reduction in pulmonary vein diameter to preoperative levels or interruption of contrast flow on follow-up CT compared with preoperative CT, and shown as ◎ in the table

The untreated, contralateral pulmonary veins are marked with diagonal lines

All six patients underwent sutureless marsupialization. The surgical materials consisted of the left atrial appendage in three cases (50.0%), bovine pericardium in two cases (33.3%), autologous pericardium in one case (16.7%), and atrial wall flap in one case (16.7%) (Table 2). Operation time was 276 ± 39 min, cardiopulmonary bypass time was 162 ± 47 min, and aortic clamp time was 110 ± 46 min. The lowest body temperature was 29.5 ± 3.6 °C. ICU stay was 3.0 ± 2.0 days, and hospital stay was 17 ± 3.3 days. All patients were discharged without major adverse events during the postoperative course.

All six patients were started on either direct oral anticoagulants (DOACs) or warfarin after surgery, and they continued to take these medications post-discharge. The average follow-up period was 62.5 ± 46.5 months.

**Fig. 3:**
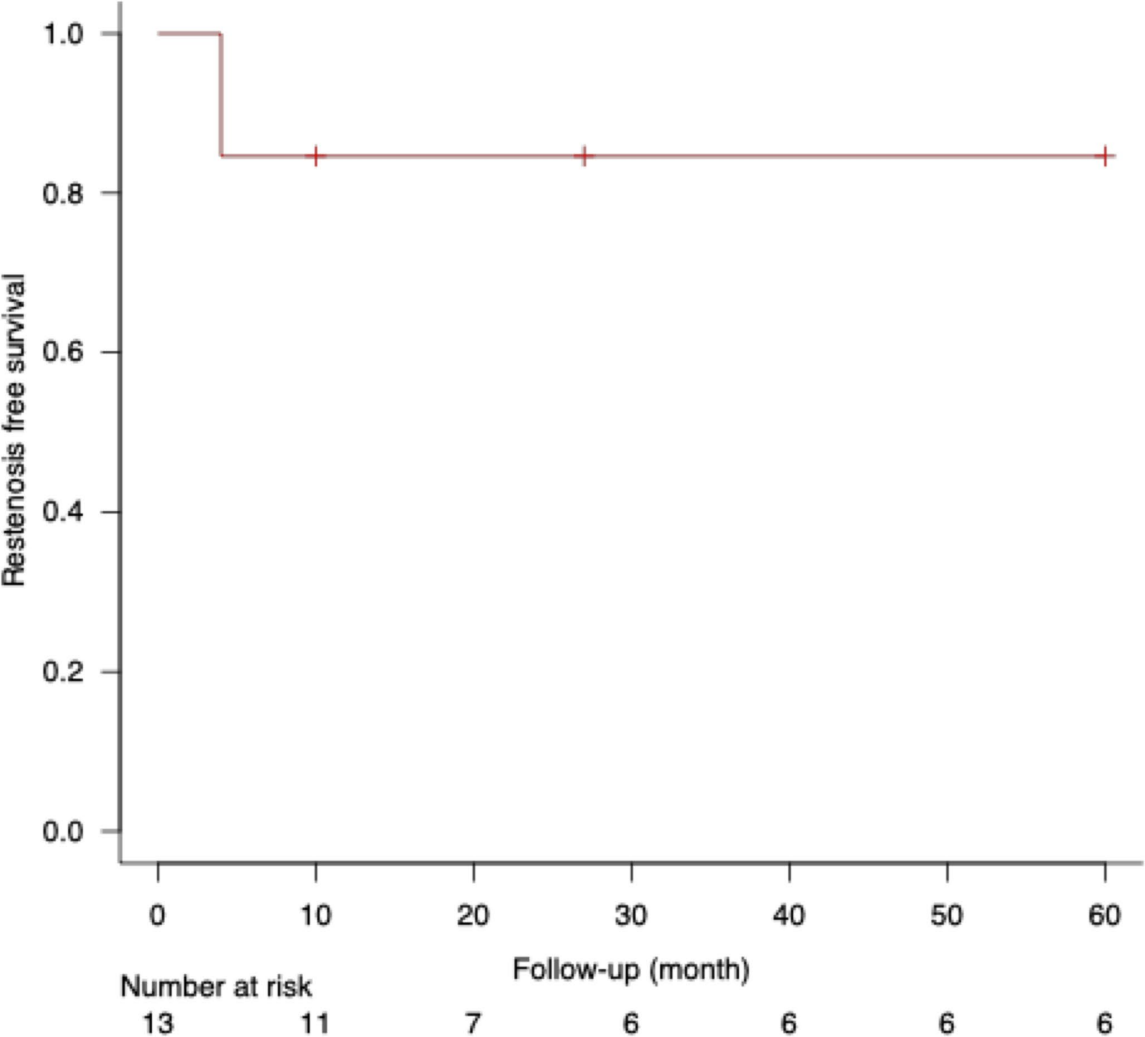
Rate of pulmonary vein restenosis based on the number of reconstructed vein. Five-year-patency is 84.6%, with a median follow-up of 62.5 ± 46.5 months

In a patient presenting with stenosis of the right and left inferior pulmonary veins (RIPV and LIPV) and occlusion of the left superior pulmonary vein (LSPV), pulmonary vein reconstruction was performed using an atrial wall flap for the right-sided veins and a left atrial appendage flap for the left-sided veins. Postoperative CT confirmed patency of all reconstructed veins; however, follow-up CT at four months showed restenoses of the RIPV and LIPV. Intraoperative findings in this case showed that, upon incision of the pulmonary vein, the intraluminal space on the peripheral side was nearly absent. This suggested that low perfusion occurred following reconstruction, which may have contributed to the development of restenosis. The patient with restenosis remained asymptomatic, and no additional interventions such as balloon angioplasty or stent placement were performed, with the case being monitored clinically. However, no restenosis was observed in the other cases. Of the 13 pulmonary veins that were surgically reconstructed, 11 remained patent in the mid-term. The 5-year patency rate for the 13 pulmonary veins treated in the present study was 84.6% (Fig. 3).

## Discussion

PVS following CA occurs in approximately 36% of patients when including mild cases (≥ 30% stenosis) [1]. Severe lesions with ≥ 70% stenosis were reported in 0.7% of patients, with only 0.1% presenting with symptoms, making it a relatively rare complication [3]. The primary clinical symptoms include dyspnea (67%), cough (45%), fatigue (45%), reduced exercise tolerance (45%), and hemoptysis (27%) [3]. Symptomatic cases with significant stenosis typically require therapeutic intervention; however, the high rate of restenosis remains a major concern in both catheter-based and surgical treatments. Fender et al. reported that, of 124 patients undergoing catheter intervention for post-ablation PVS, the 3-year restenosis rates were 49% with balloon angioplasty alone, 25% with stenting, and 37% overall, underscoring the challenge of durable outcomes [3]. Similarly, Schoene et al. reported a 38% rate of restenosis within five years following surgical pulmonary vein reconstruction using bovine pericardium or polytetrafluoroethylene patches [5].

One proposed mechanism of restenosis following surgical repair is direct suturing to the pulmonary vein wall, which may induce turbulent flow, fibrous neointimal proliferation, and anatomical distortion at the pulmonary vein-left atrial junction [6]. To overcome this, we used the sutureless marsupialization technique, originally developed for the surgical treatment of total anomalous pulmonary venous connection in pediatric cardiac surgery. This approach avoids direct suturing to the pulmonary vein wall. The procedure involves incising the left atrium across the stenotic segment of the pulmonary vein, then covering the opened area with autologous tissue such as the left atrial appendage or pericardium to create a new anastomosis between the pulmonary vein and left atrium. By minimizing surgical trauma and scar formation, this technique is expected to improve postoperative vessel patency. In our institution, we have achieved good early outcomes with the use of the left atrial appendage, consistent with previously reported cases that also demonstrated early vessel patency [7].

In the present study, the sutureless marsupialization technique was applied to 13 pulmonary vein lesions (including both stenosis and total occlusion) in six patients. The 5-year vessel patency rate was 84.6%, which compares favorably with outcomes of existing catheter-based and conventional surgical treatments. Restenosis was observed in two lesions, both of which were stenotic (as opposed to occluded). In these cases, the intraluminal space distal to the stenosis could not be adequately visualized, and sufficient peripheral runoff could not be achieved, likely contributing to the postoperative occlusion. Conversely, in cases of complete occlusion, vessel patency was maintained postoperatively when a residual lumen was preserved distal to the lesion. Although preoperative CT showed complete obstruction in the patient No. 2, No.3, No. 4, and No. 6, there were no significant differences in CT findings among them. In the patient No. 6, however, no lumen or distal blood flow was visible intraoperatively, making reconstruction unfeasible. These findings indicate that determining treatment eligibility based solely on preoperative imaging is difficult; intraoperative assessment remains essential for final surgical decision-making. We believe that the key intraoperative finding for successful reconstruction is confirming that the intraluminal space of the pulmonary vein is adequately preserved beyond the stenotic lesion. Further investigation is needed to determine whether modalities such as pulmonary perfusion scintigraphy or pulmonary angiography may offer useful preoperative examinations.

To ensure long-term patency, anticoagulation therapy and the appropriate selection of covering materials are critical. Although half of the patients presented with hemoptysis preoperatively, all symptoms resolved after surgery. Because postoperative anticoagulation is considered important for maintaining long-term patency, we initiated it while closely monitoring for recurrent hemoptysis. In addition, our institutional policy favors the use of autologous tissue, which we believe contributes to good long-term outcomes. Wakabayashi et.al also reported the use of the left atrial appendage can reduce the risk of thromboembolism due to AFib, since the left atrial appendage is the most common site for cardiac thrombus, especially in case of left lesion [9]. The use of synthetic grafts or xenografts has been associated with restenosis due to neointimal proliferation even in non-sutureless procedures, suggesting that material choice significantly impacts patency [7, 9–12]. Assessment of further cases will be necessary to evaluate patency outcomes by material type.

To date, reports of the use of sutureless marsupialization for acquired PVS/PVO have largely been limited to case reports [7, 9–12]. The present study is the first to evaluate mid-term outcomes. With the increasing number of catheter ablations, the number of PVS cases requiring surgical intervention is expected to grow. The present findings demonstrate good results compared with previously reported catheter-based and conventional surgical therapies. In the absence of established treatment guidelines, this study may contribute to future decision-making strategies for managing acquired PVS.

This study has several limitations that warrant consideration. First, the sample size was relatively small, involving only six patients who underwent surgical treatment. This limits the statistical power and generalizability of the findings. Second, this was a single-center study, which may introduce selection bias and restrict the broader applicability of the results. Third, though this study reports mid-term outcomes, longer-term follow-up data are necessary to definitively assess the durability of the sutureless marsupialization technique. Fourth, further cases are required to evaluate patency outcomes based on the different types of covering materials used (e.g., left atrial appendage, autologous pericardium, bovine pericardium, left atrial wall), since the current sample size was insufficient for this analysis. Finally, though preoperative imaging is crucial for diagnosis, our experience indicates that determining treatment eligibility based solely on preoperative assessment is challenging, and intraoperative findings remain essential for final surgical decision-making. The difficulty in precisely predicting outcomes or selecting cases purely preoperatively can be considered a limitation of the current approach. Further studies should assess whether modalities such as pulmonary perfusion scintigraphy or pulmonary angiography can improve preoperative evaluation and guide treatment selection.

## Conclusion

In this study, the mid-term outcomes of the sutureless marsupialization technique for acquired PVS and PVO following CA for Afib were evaluated. The results demonstrate feasible mid-term vessel patency, with a 5-year patency rate of 84.6% for the surgically reconstructed pulmonary veins. To the best of our knowledge, this study provides the first report of mid-term outcomes for the application of the sutureless marsupialization technique in acquired PVS post-catheter ablation. Given the expected increase in PVS cases requiring intervention, the present findings offer valuable data and may contribute to guiding future treatment strategies for this challenging complication.
